# Different Shades of Kale—Approaches to Analyze Kale Variety Interrelations

**DOI:** 10.3390/genes13020232

**Published:** 2022-01-26

**Authors:** Christoph Hahn, Nicholas P. Howard, Dirk C. Albach

**Affiliations:** 1 Institute for Biology and Environmental Sciences, Carl von Ossietzky University Oldenburg, 26111 Oldenburg, Germany; howar622@umn.edu (N.P.H.); dirk.albach@uol.de (D.C.A.); 2 Fresh Forward Breeding & Marketing, 4024 BK Eck en Wiel, The Netherlands

**Keywords:** *Brassica oleracea*, kale, cabbage, collards, Illumina *Brassica* 60K array, SNP marker, SPLoSH, shared haplotype information

## Abstract

*Brassica oleracea* is a vegetable crop with an amazing morphological diversity. Among the various crops derived from *B. oleracea*, kale has been in the spotlight globally due to its various health-benefitting compounds and many different varieties. Knowledge of the existing genetic diversity is essential for the improved breeding of kale. Here, we analyze the interrelationships, population structures, and genetic diversity of 72 kale and cabbage varieties by extending our previous diversity analysis and evaluating the use of summed potential lengths of shared haplotypes (SPLoSH) as a new method for such analyses. To this end, we made use of the high-density *Brassica* 60K SNP array, analyzed SNPs included in an available *Brassica* genetic map, and used these resources to generate and evaluate the information from SPLoSH data. With our results we could consistently differentiate four groups of kale across all analyses: the curly kale varieties, Italian, American, and Russian varieties, as well as wild and cultivated types. The best results were achieved by using SPLoSH information, thus validating the use of this information in improving analyses of interrelations in kale. In conclusion, our definition of kale includes the curly varieties as the kales in a strict sense, regardless of their origin. These results contribute to a better understanding of the huge diversity of kale and its interrelations.

## 1. Introduction

Domestication requires an initially large variation in traits from which farmers can select. However, the variation in numerous traits, such as disease resistance or tolerance to environmental conditions (heat, cold), is often lost during breeding and domestication [1]. Selection for yield and emphasizing quality traits have often been favored over originality and diversity. The decline of diversity in crop species is also hastened by new generations of growers and modern breeding techniques emphasizing uniformity in crops and, thus, old landraces are continuously lost [2]. The breeding of improved genotypes will, nevertheless, depend on using the available genetic diversity, especially in the face of climate change. Such diversity could be exploited in rare or underutilized landraces; however, the nature of this diversity is not well understood.

For *Brassica oleracea* (2*n* = 18), a broad range of genetic and morphological diversity exists. Its evolutionary history and domestication origin is still widely unknown [3]. Current knowledge indicates the Eastern Mediterranean as the center of domestication for *B. oleracea* [3,4,5], either by early cultivated forms originally from the Atlantic coast brought to this region [6], or by in situ domestication of Mediterranean wild species [7]. Studies of Schiemann [8], Lizgunova [9], and Mei, et al. [10] specifically report Sicily as the geographic origin. The recent analyses of Mabry, et al. [3] support *B. cretica* as the closest living wild relative to *B. oleracea*.

Cultivars are mainly grouped based on their edible structure, including cabbages, kohlrabi, broccoli, cauliflower, romanesco, Brussels sprouts, and kale. Especially the latter one, kale, being prominent especially in Northern European cuisine in wintertime for hundreds of years, has been in the spotlight globally in recent years. It has been put on the growing list of foods considered “superfoods” due to its various health-benefitting compounds. There are several metabolites contributing to this rating, primarily glucosinolates, carotenoids, and flavonoids, as well as several vitamins (including vitamin C) and mineral nutrients [11]. Since the composition and amounts of these ingredients differ according to variety and landrace, there is a huge demand to preserve the diversity as a source for new breeding approaches aiming to optimize the nutritional content of varieties. A key aspect for the breeding of improved varieties will be to exploit the potential offered by rare and mostly underutilized landraces. In addition to a large number of such valuable local landraces in Northern Germany (East Frisia) based on a long-lasting tradition with kale, there is a long list of different kale varieties available from other areas as well. They differ in growth, habit, leaf color, leaf characteristics, and nutritional content [11].

**Figure 1:**
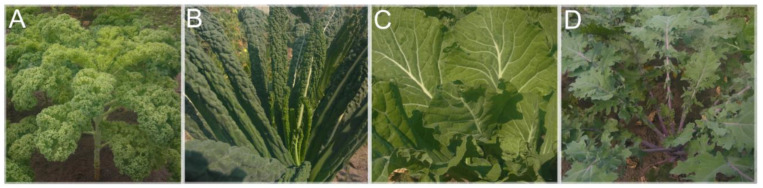
Different types of kale cultivars. (**A**) Curly kales, (**B**) Italian kales, (**C**) collards primary found in the USA, and (**D**) Russian or Siberian kales.

According to origin, one can distinguish between at least three different types of kale cultivars: curly kales, Italian kales, and collards [12] (Figure 1). Curly kales (Scotch type, *Brassica oleracea* convar. *acephala* var. *sabellica*) are to be considered as kale in the strict sense and are mostly grown in Northern Europe. Italian kale varieties of the Lacinato type (*Brassica oleracea* convar. *acephala* var. *palmifolia*) have dark green savoyed blade shape leaves and are originally grown in Tuscany and have a long tradition in Italian cuisine. The third group, collards (*Brassica oleracea* convar. *acephala* var. *viridis*), are mainly found in the Southern part of the United States. They are characterized by large and flat roundish leaves and most resemble wild and feral cabbages, which are big-leaved herbs. A fourth group, Russian kales (Siberian type, *B. napus* var. *pabularia*) (Figure 1) have been domesticated from a related but different species. Despite the wide distribution and morphological diversity, to date, there has not been a strong focus on kale in the *Brassica* research area. Studies on Turkish kale populations [13], kale cultivars and landraces from Europe [14,15], wild and cultivated populations of leafy kale in Italy [16], collards in the USA [17,18], and a recent study on the vegetative domestication syndrome of kale [19] are sole exceptions. Extensive insights into interrelationships in kale are crucial, given the huge diversity of varieties, not only considering the cross-compatibility of kales with all other varieties of *Brassica oleracea*, but also concerning studies demonstrating that closely related cultivars are phytochemically more similar to each other [12]. Interrelationship information helps in predicting phytochemical but also physiological (notably frost resistance [20]) responses and aids the conservation of genetic resources.

In the past, *Brassica* research assaying genetic variation has been conducted using different marker systems. Most studies in the early 21st century on *B. oleracea* genetic diversity commonly applied simple sequence repeats (SSRs, also referred to as microsatellites) [10,21,22,23,24,25,26]. In the most recent years, microsatellites have been increasingly replaced by single nucleotide polymorphisms (SNPs) as the marker of choice in population genetics studies of crop species [27]. They are the most common type of genetic polymorphism and unlike SSRs, are distributed throughout the genome in both coding and non-coding DNA regions [28,29]. Additionally, while biallelic, SNP data can be made more informative by considering haplotype data, which can create multi-allelic information that thus considers linkage (closely neighbored DNA sequences on a chromosome are inherited together). SNP detection can either be performed through traditional sequencing or by using genotyping arrays. SNP genotyping platforms generally are more powerful in detecting structure and provide information on far more markers than is typical with SSR data sets [29,30]. Thus, SNPs have become the preferred marker system for discriminating between cultivars and have been applied in various studies on *Brassica* crops, e.g., for *B. napus* [31], *B. juncea* [32], *B. rapa* [33,34], and *B. oleracea* [12,18,33,35].

Our previous research into kale diversity [12] was made using the fluorescence-based KASP SNP genotyping platform (Kompetitive Allele Specific PCR) [27,36] with 100 KASP markers well distributed over the *B. oleracea* genome. Our results allowed us to clearly distinguish between major groups of kale cultivars: curled varieties, Italian varieties, and American kales. They were found to be distinct from non-kale *B. oleracea* cultivars and a wild (feral?) cabbage cultivar. Furthermore, we could widely confirm that the wild cabbage is the ancestor of cabbage and kale varieties, fitting with the current *Brassica* phylogeny reported by Arias and Pires [37]. KASP assays are flexible, cost-effective, and fast [38]. Nevertheless, the application to kale data revealed some ambiguities and limitations. The number of markers was limited, they were considered unlinked and did not cover the full genome. The relationships between certain varieties remained doubtful and others unresolved. Support values were generally low. Thus, these limitations necessitate a different approach to better understand the relationships among kale cultivars. In 2016, Clarke, et al. [39] published the recently developed *Brassica* 60K Illumina Infinium SNP array, consisting of 52,157 SNP markers from the A genome (*B. rapa*, 2*n* = 20) and the C genome (*B. oleracea*, 2*n* = 18). It was designed based on genomic data of *B. napus* (AACC, 2*n* = 38), the hybrid between *B. oleracea* and *B. rapa* [40]. Since then, several studies have applied this array for analyses in *B. napus* [41,42,43,44,45,46,47,48,49,50,51,52,53], among others and *B. oleracea* [18,54,55,56], but there have been no published studies focusing on kale that used this array.

The presence of large genotyping arrays allows for high-resolution analysis and the description of genetic relationships of individuals [57]. Higher fidelity of relationships is theoretically possible when considering more markers while accounting for linkage via the identification of shared haplotype data between pairs of individuals. A shared haplotype is defined as a series of adjacent SNP alleles that are held in common between a pair of individuals in a population. This concept has recently been used for pedigree reconstruction in apple (*Malus* × *domestica*) and cherry (*Prunus cerasus*) [58]. The authors reported this as a solution to address some limitations in the exclusive use of unlinked SNP data for estimating relationships between pairs and groups of individuals. Analogously, kale varieties that are more closely related should have more haplotypes in common than those that are not. Several linkage maps have been published for *B. oleracea* [54,56,59,60,61,62] that used the *Brassica* 60K SNP array, which could enable this sort of analysis. However, no genetic map is available to date for kale using SNPs from the 60K array. Thus, the closest maps relevant for such an analysis are those for cabbage (*B. oleracea*, C genome) or rapeseed (*B. napus*, A + C genome). The incorporation of linkage information via haplotype data for characterizing kale, which is distinguished by considerable crossbreeding, should provide higher resolution results in comparison to solely using individual SNP data.

In this paper, we use SNP data and the summed potential lengths of shared haplotype (SPLoSH) information [58] to investigate the interrelationships of kale and cabbage varieties by extending our previous diversity analysis [12]. We include additional varieties and evaluate a new method for such analyses. To improve the kale diversity analysis (i) we made use of the high-density *Brassica* 60K Illumina Infinium SNP array [39]; (ii) we compiled a subset of those SNPs matching SNPs included in the genetic map of Wu, et al. [48]; (iii) we generated SPLoSH information, as described in Howard, et al. [58], between every pair of individuals included in our study using the genetic map from Wu, et al. [48] and used this data to infer the relative relatedness between kale cultivars; and (iv) we compared the different approaches of analysing kale diversity to demonstrate the advantages of using SPLoSH information in such analyses.

## 2. Materials and Methods

### 2.1. Genotyping Kale and Cabbage Samples Using the Brassica 60K Illumina SNP Array

#### 2.1.1. Plant Material

For this study, 69 different kale and cabbage varieties and three accessions of *Brassica* species (Table 1) were grown at the botanical garden of the Carl von Ossietzky University, Oldenburg. Seeds were germinated in a greenhouse under natural daylight and at the 5-leaf-stage were transplanted outside into a field. Water and fertilizer were applied according to standard cultivational practices. Fresh leaf material was collected from the full-grown plants, subsequently silica dried (plastic bag with silica beads), and then prepared for further analysis by cutting leaf discs from the plant material using the LGC plant sample collection kit (KBS-9370-001). Voucher specimens of kale varieties are deposited at the herbarium of the Carl von Ossietzky University, Oldenburg (OLD).

#### 2.1.2. Genotype Data

All samples were genotyped on the high-density *Brassica* 60K Illumina Infinium SNP BeadChip array [39], which was outsourced to TraitGenetics GmbH (Gatersleben, Germany), including DNA extraction. Samples were processed according to the manufacturer’s protocol (Infinium HD Assay Ultra Protocol Guide; Illumina [63]). This encompassed a series of steps (such as DNA amplification, hybridization of sample DNA onto the bead chip and imaging of the bead assay) described in detail by Gunderson, et al. [64]. Illumina GenomeStudio software (2015 v2.0.4; Illumina, Inc., San Diego, CA, USA) was used to cluster and visualize SNP array data with default clustering parameters for further analysis.

#### 2.1.3. Processing of Data and Quality Filtering

Several quality filtering steps were performed on the 52,157 SNPs in the array. We removed the following SNPs from the dataset: (i) SNPs that had >2% missing data; (ii) SNPs that did not have two homozygous groups; (iii) SNPs that were not polymorphic (i.e., showing identical genotype calls across all samples); and (iv) SNPs that were likely to have null alleles. SNPs with likely null alleles were identified by those with a distribution of Norm R values greater than 0.09. Quality filtering was done using GenomeStudio and Microsoft Excel (v.16.0., 2019). The dataset is called the “filtered dataset” in the following (Supplementary File S1).

**Table 1:** Information on kale and cabbage varieties included in this study. For detailed information on accessions and variety names refer to Supplementary Table S1. We generally followed the classification by Gladis and Hammer [65].

Accession	Group (Origin)	Phenotypic Grouping	Additional Information
GER 1–26	German	curly	
OSTFR 1–10	East Frisian	curly	
ITAL 1–13	Italian	Lacinato-type	
ITAL 14–16	Italian	wild, non-Lacinato	native to Sardinia, Elba island, and Calabria
USA 1–7	American	non-curled collards	USA 5: American farmer-bred curly kale
BRASS 1–2	Russian	lobed/frilled	
BRASS 3–5	wild Brassica		
BOLERA 1–15	non-kale *B. oleracea*		

### 2.2. Employing Linkage Information Using a Published B. napus Genetic Map

In addition to the high-quality filtered SNP set described above, information from a previously published *B. napus* map [48] was used to incorporate linkage information into the diversity analyses. The decision to use a *B. napus* map was based on the high percentage of SNP markers from their map that are held in common with our filtered dataset. For the available *B. oleracea* maps the concordance was much lower or could not be evaluated adequately due to marker name changes. The 15,622 SNPs of the chosen map were filtered to put only those SNPs that were in our filtered dataset into a new dataset (“map dataset” in the following; Supplementary File S2). 

Summed potential lengths of shared haplotypes (SPLoSH) information was generated using the Python script HapShared [58] and with cM positions for the SNPs from the map dataset (based on the genetic map from Wu, et al. [48]) as a way to incorporate linkage information into the diversity analyses. SPLoSH information has been previously used to conduct pedigree reconstruction in apple and cherry [58] and was used here to estimate the level of shared haplotypes between each pair of individuals as a metric for genetic distance. The generated SPLoSH information (Supplementary File S3) was used in downstream analyses as described below. A threshold of 30 cM was used for the generation of SPLoSH information.

### 2.3. Data Analysis

For downstream analyses described in the following, we used (i) the filtered dataset; (ii) the map dataset; and (iii) the SPLoSH information. 

To determine relationships between the sampled individuals and to test putative sample grouping, we used different methods to analyze the data. First, the data were subjected to principal component analysis (PCA) using the package stats in the statistical software R v.4.1.1 [66] (Supplementary File S4). Prior to analysis, a Euclidean distance matrix was computed based on the data, and missing values of the dataset (distance between an individual and itself) were imputed with the principal components analysis model implemented in the package missMDA [67]. Data were scaled, and the results of the PCA were displayed with the R package ggplot2 [68] using the PCA score plot for the first two principal components with each point in the plot representing a sample. 

Second, a discriminant analysis of principal components (DAPC) was conducted using the corresponding function implemented in the R package adegenet [69] (Supplementary File S4). The optimal number of PCs to retain was identified using the *a*-score as described in the corresponding package vignette. All eigenvalues (discriminant functions) were retained. As a result, a DAPC-scatterplot was produced. Further, the DAPC object included the population membership probability for all samples that could be illustrated with a compoplot (composite stacked bar plot) to exploit group memberships. 

To assign individuals to population clusters and to determine genetically distinct groups, we used the software STRUCTURE v.2.3.1 [70,71,72] (except for the SPLoSH information, see discussion). First, the “infer lambda” option was run for *K* = 1 with five independent iterations to infer the optimal value for lambda fitting the data. For the subsequent runs, the admixture ancestry and independent allele frequencies models without population affiliation were chosen, leaving the other parameters at default. For values of *K* (the putative number of genetic groups) from 2–6, 20 independent analyses with a run length of 1,000,000 MCMC generations were performed, each with a burn-in period of 100,000 replications. The preferred value of *K* was determined with the Δ*K* method by Evanno, et al. [73] using the CLUMPAK webserver (“Best K” feature) [74]. The preferred number of *K* clusters was the one with the highest Δ*K* value. Runs were averaged using CLUMPP [75] with the LargeKGreedy algorithm and the G’ pairwise matrix similarity statistics and results were visualized with DISTRUCT [76], both implemented in CLUMPAKs “main pipeline” feature.

For illustrating evolutionary history, first a phylogenetic network was calculated using the neighbor-net method [77] implemented in SplitsTree v.4.13.1 [78] with the following parameters: uncorrected P-distances, edge weights estimated using ordinary least squares variance, and using the equal angle algorithm [79] with equal-daylight and box-opening optimization (5 iterations each) [80]. Ambiguous states were matched.

In addition (except for the SPLoSH information, see discussion), a phylogenetic tree of the kale samples was constructed with MrBayes v.3.2.7 [81] using the GTR+I+Γ model. The program was run for 10,000,000 generations, trees sampled every 1000 generations, with eight simultaneous chains (two independent runs of four chains each—three heated, one cold). The first 25% of the generations were discarded as burn-in. All parameter values were estimated during the analysis. Base frequencies and rate matrix have Dirichlet prior. Stationarity was assumed when the potential scale reduction factor (PSRF) reached one and the average standard deviation of split frequencies between independent runs approached 0. A majority-rule consensus of the remaining trees from the two runs was produced with posterior probabilities.

## 3. Results

After employing the quality control and filtering measures, a total number of 6167 SNPs were retained in the filtered dataset for further analysis. From this set of 6167 SNPs, there was 0.94% missing data (with a max of 52.1% missing data in the *B. rapa* sample). For the map dataset, 2668 SNPs were extracted matching the genetic map from Wu, et al. [48] (1.07% missing data; 74.4% max (*B. rapa*)). A heatmap of SPLoSH values demonstrated the genetic relatedness between every pair of samples included in this study, highlighting closely and distantly related varieties (Supplementary Figure S1).

**Figure 2:**
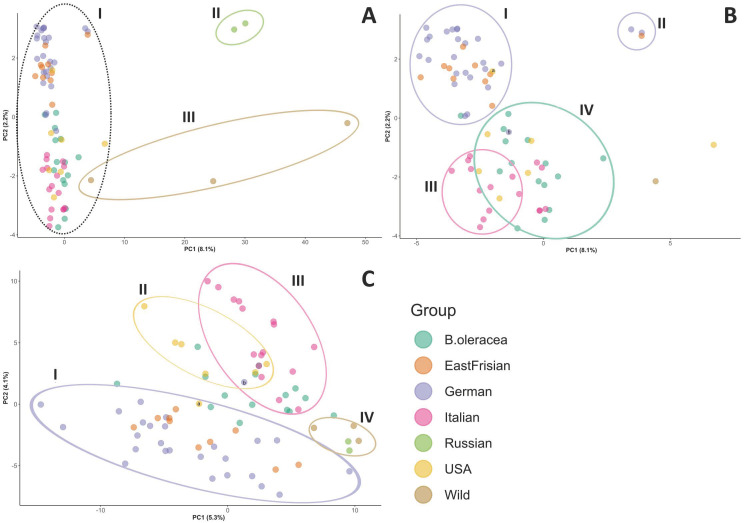
Principal components score plot showing PC 1 and PC 2 of PCA results for the samples obtained from (**A**) + (**B**) SNP data of the filtered dataset, and (**C**) using the SPLoSH information. The proportion of the total variance that is explained by the first (PC 1) and second (PC 2) principal component is indicated in brackets. Part (**B**) is a magnification of the dotted cluster. Details on the observed clusters (Latin numbers) are explained in the text. Each point represents one sample. Plots labelled with sample names are provided as Supplementary Figure S3. “a” = Olympic Red, “b” = Dutch kale.

The structure of the samples was visualized by principal component analysis (PCA) score plot (Figure 2). For the filtered dataset, principal component (PC) 1 explained 8.1% of the genetic variation of the data and principal component 2 explained 2.2% (Figure 2A,B). For the SNPs of the map dataset, PC 1 explained 7.4% of the genetic variance and PC 2 explained 3.6% (Supplementary Figure S2). Using the shared haplotype information (SPLoSH), PC 1 explained 5.3% and PC 2 explained 4.1% (Figure 2C). Considering the SNPs of the filtered dataset, the outgroup (Cluster III: wild *Brassica*, and Cluster II: Russian kales (*B. napus*)) was separated from all other samples (Cluster I) (Figure 2A). Looking deeper into Cluster I, four minor clusters appeared: The curly kales (German and East Frisian) were nearly perfectly separated from the others (Cluster I and II in Figure 2B). The three varieties in Cluster II (i.e., “Lerchenzungen”, “Unterweser”, and “Neuefehn”) were separated from the remaining curly kales. Variety “Olympic Red” (labelled “a” in Figure 2B) originated in the USA but is of curly shape and clustered within the curly kales. Cluster III (Figure 2B) was made up of Italian varieties of the Lacinato type (cf. group affiliation in Table 1). Cluster IV was not that clearly defined and contained all other American kales together with the remaining Italian kale varieties (of non-Lacinato type) as well as the other *B. oleracea* cabbages. Regarding the PCA based on the SNPs from the map dataset (Supplementary Figure S2), no clear cluster pattern could be recognized. Using SPLoSH information instead of SNP data (Figure 2C) contributed to clearer cluster separation. The German and East Frisian curly kales were separated from the other samples along PC 2 (Cluster I). The three curly kales mentioned before and formerly grouped into a separate cluster were now integrated into the other curly varieties in Cluster I. Cluster II covered the USA kale varieties, but not exclusively. Cluster III mainly contained the Italian varieties. The Italian kales and American kales were separated from each other along PC 1 and from the curly varieties along PC 2. Cluster IV was made up of the outgroup samples, i.e., the Russian kale varieties and the wild *Brassica* samples. The non-kale *B. oleracea* samples did not cluster together but were distributed along the PC 1 axis. Because the high percentage of missing data for the Russian kale and wild samples could inflate SPLoSH values, influencing clustering, clustering analyses with SPLoSH information was also conducted with this material excluded. The results were similar.

**Figure 3:**
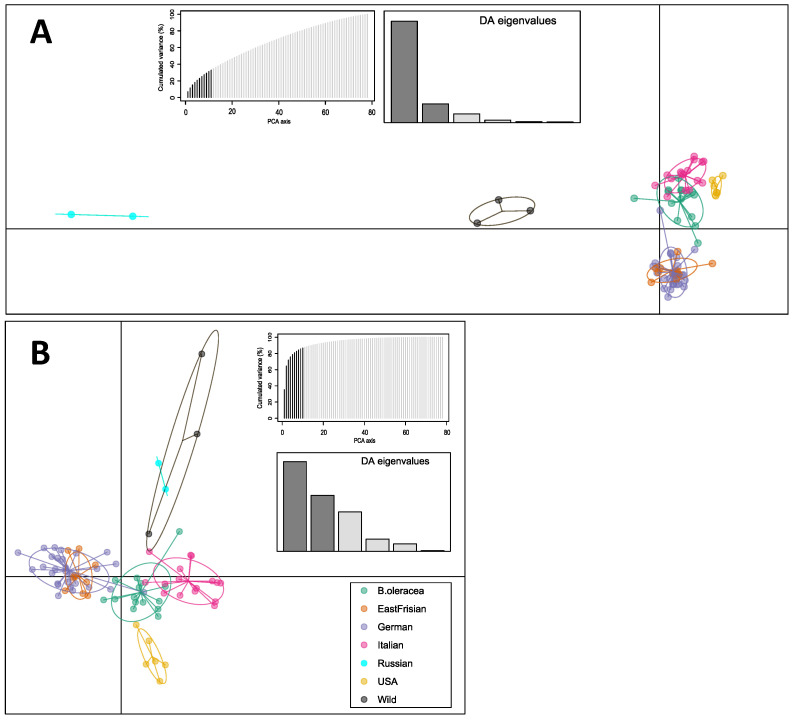
Discriminant analysis of principal components (DAPC). Scatterplot showing the first two principal components for the samples obtained from (**A**) SNP data of the filtered dataset, and (**B**) using the SPLoSH information. Dots represent individual samples; clusters are marked with ellipses. Graphs of the PCA and DA eigenvalues retained are shown. Plots labelled with sample names are provided as Supplementary Figure S4.

Based on the optimized *a*-score from discriminant analysis of principal components (DAPC), the number of PCs chosen to be retained were 11 for the filtered dataset, 13 (map dataset) and 10 (SPLoSH information), respectively. Results for the filtered dataset (Figure 3A) showed that PC 1 separated the wild *Brassicas*, the Russian kales, and the American kales from all other samples, and the PC 2 separated the curly kale varieties (German and East Frisian), Italian kales and the non-kale cabbages. German and East Frisian kales clustered together, building a separate group. Concerning the SNPs from the map dataset the patterns became blurrier (Supplementary Figure S5). Instead, although marginally, the use of SPLoSH information (Figure 3B) gave the clearest separation, as the results showed five distinct and separate clusters, i.e., all German and East Frisian curly kales, American kales, Italian kales, non-kale *B. oleracea*, and the wild *Brassicas* together with Russian kales. 

**Figure 4:**
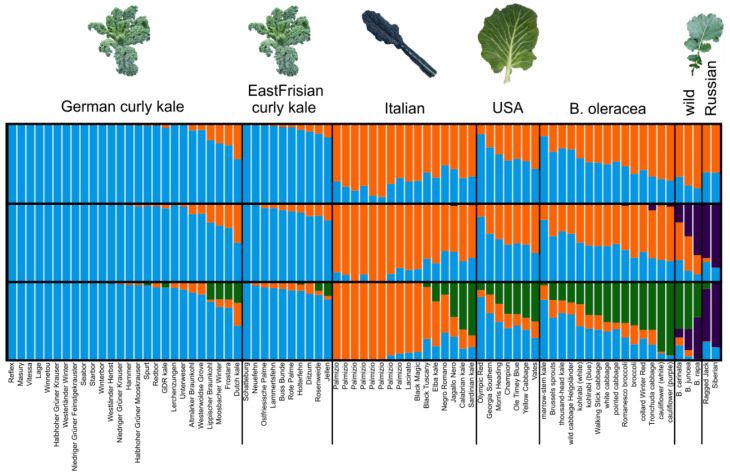
STRUCTURE plot for kale and cabbage varieties from different origins for *K* = 2–4, based on the SNP data from the filtered dataset. Each column represents one variety, colors indicate the proportion of membership to different clusters. Each figure is a combination of 20 replicates.

In the STRUCTURE analyses, only the results from the independent model are shown because both the independent and correlated allele frequency models were similar. *K* values from 2–4 were reported (Figure 4) with Δ*K* indicating *K* = 2 as the optimal cluster number. Regarding the filtered dataset (Figure 4), a clear differentiation between the curly kales (German and East Frisian) and most other varieties was found. The most precise differentiation between the varieties could be seen at *K* = 4. Apart from the curly kales, Lacinato-type Italian varieties and Russian kales formed clusters, whereas the American varieties together with the wild Italian ones (non-Lacinato type, cf. Table 1) and the non-kale cabbages formed genotypes gradually diverging from the other three types with cauliflower and broccoli being most distinct. The wild *Brassica* samples, moreover, had the highest membership probabilities with the Russian kales. Concerning the map dataset (Supplementary Figure S6), the groups were less clearly separated and more heterogeneous.

The DAPC genotype composition plots (compoplots) (Supplementary Figure S7) revealed the membership probabilities of certain varieties to different clusters in more detail, i.e., some of the kales treated as German had a higher probability to be grouped within the East Frisian kales (“Altmärker Braunkohl”, “Westerwoldse Grove”, “Lippischer Braunkohl”), and, vice versa, some East Frisian kales (“Neuefehn”, “Rosenweide”) were more likely to belong to the German cluster. Non-kale cabbages “thousand-head kale”, “wild cabbage”, “pointed cabbage”, and “white cabbage” showed substantial membership probabilities of the American kale cluster (Supplementary Figure S7B). *Brassica carinata* had a probability of more than 50% of membership in the *B. oleracea* cluster (Supplementary Figure S7C). 

**Figure 5:**
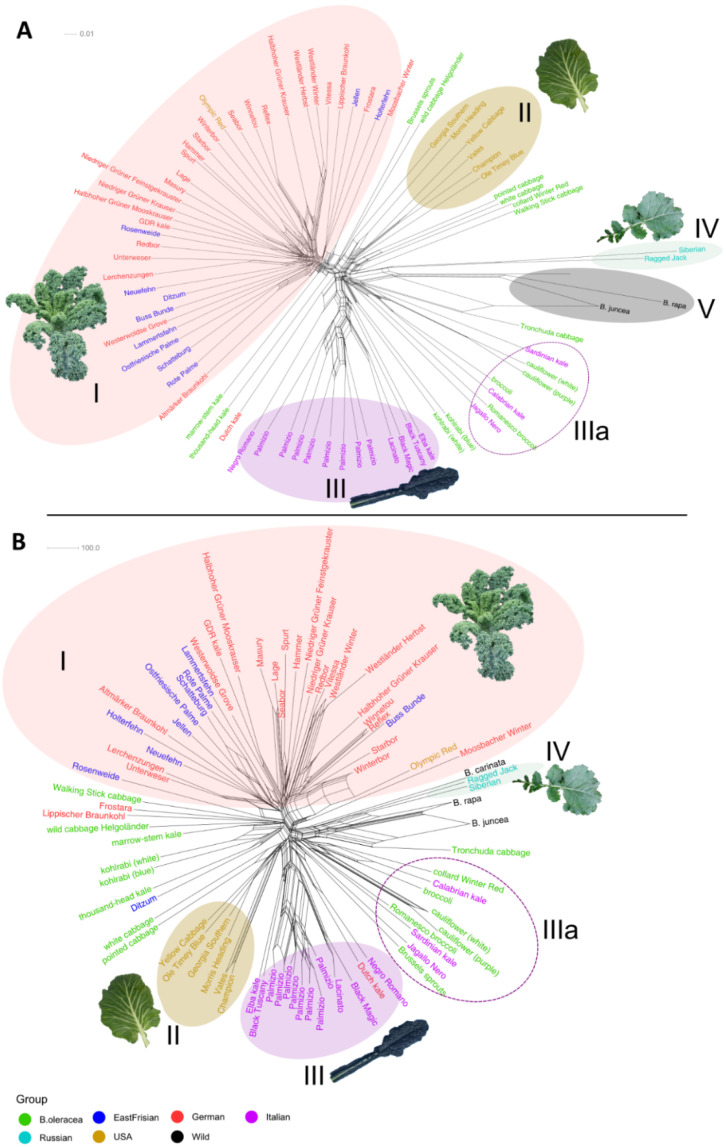
Neighbor-net showing genetic distances of different kale and cabbage varieties, based on (**A**) SNP data from the filtered dataset, and (**B**) using the SPLoSH information. Sample names are color coded according to their origin. Details on the observed clusters (Latin numbers) are explained in the text.

From the neighbor network based on the filtered dataset (Figure 5A), five clearly defined clusters could be recognized. All curly kale varieties (German and East Frisian) made up Cluster I. The only variety that did not fall directly into this cluster was the Dutch Kale. The “Olympic Red” kale, although originated in the USA, was, as previously mentioned, of curly shape. It was seen that the East Frisian kales “Schatteburg”, “Ostfriesische Palme”, and “Rote Palme” clustered together; same did “Lerchenzungen” and “Unterweser” (see discussion section) as well as “Niedriger Grüner Feinstgekrauster” and “Niedriger Grüner Krauser”, and “Halbhoher Grüner Mooskrauser” and “GDR kale”. The American collard kales were all clustered together as Cluster II, neighbored by different non-kale *B. oleracea* cultivars, among them the wild cabbage. Equally, the Italian kales of the Lacinato type formed Cluster III. The wild Italian kales were distributed among the *B. oleracea* cultivars of Italian ancestry (cauliflower, broccoli and romanesco) (Cluster IIIa). The Russian kales (Cluster IV) were found near the wild *Brassicas* (Cluster V) which were considered the outgroup taxa. Regarding the SNPs from the map dataset (Supplementary Figure S8), no clear clustering was apparent. All varieties from the different origins were distributed together without any noticeable pattern. The neighbor network built using SPLoSH information was similar to that of the filtered dataset (Figure 5B). Most of the curly kales clustered together (Cluster I), so did the American (Cluster II), Russian (Cluster IV), and Italian kales (Cluster III). For the Italian varieties, the same picture appeared as described above: The wild Italian kales together with the Italian-derived cabbages formed an “Italian subcluster” IIIa. The other non-kale *B. oleracea* cultivars did not form their own separate cluster, although they were found near to each other neighboring Clusters I and II.

**Figure 6:**
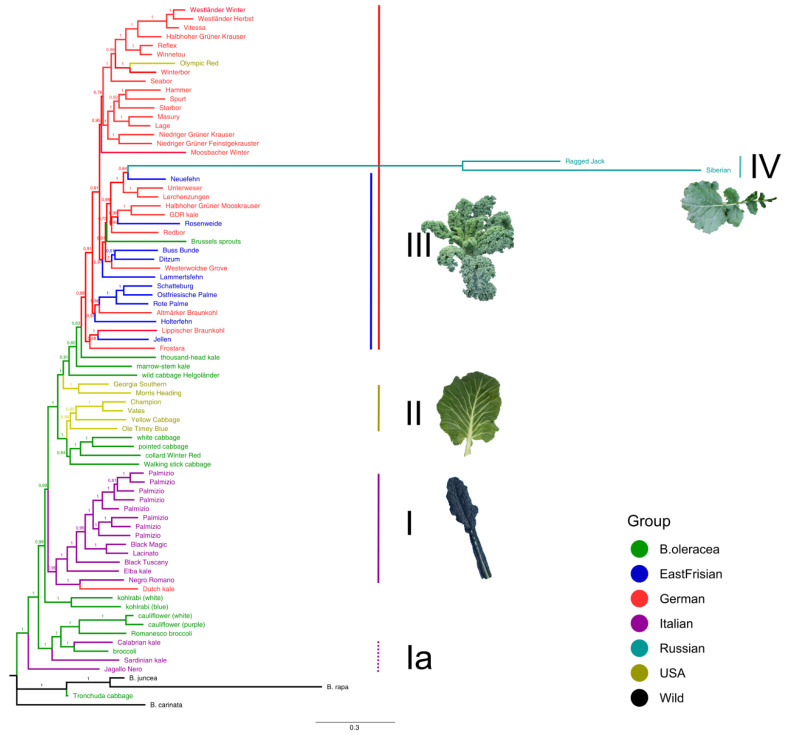
Bayesian inference phylogeny of kale and cabbage varieties, based on the SNP data from the filtered dataset. Sample names are color coded according to their origin. Numbers above branches are Bayesian posterior probabilities.

The same groups and clusters as described before could be consistently identified from the Bayesian inference phylogenetic tree based on the filtered dataset (Figure 6). The Italian kales were located close to the outgroup; Lacinato-type forming one clade (I) and the wild ones together with Italian-derived cabbages forming another clade (Ia). The American kales (II) were found together with the other non-kale *B. oleracea* cultivars, directly neighbored by wild cabbage “Helgoländer”. The topmost varieties in the tree were the group of curly kales (III), including the curly American variety “Olympic Red”. Nested within this curly kale clade were the Russian varieties (IV), however, this position was not well supported. Concerning the phylogenetic tree constructed from the map dataset with many fewer SNPs, the resolution was considerably worse (Supplementary Figure S9). The branches were very short and nodes were hardly visible. Samples with similar origins were not grouped together with any noticeable pattern. Furthermore, the support of the branching was, in general, quite low.

## 4. Discussion

In the present study, the population structure and genetic diversity of 69 kale and cabbage cultivars and three *Brassica* species were assessed using data obtained from the high-density *Brassica* 60K genotyping array. We explored a new method to analyze this data by using shared haplotype information (SPLoSH) as a measure of genome-sharing among the individuals that emerged as highly useful for improving the mapped SNP data. We were able to make statements about the clustering and individual relationships of the studied kale and cabbage varieties, such as the differentiation between curly kales and the cultivars from Italy, North America, and Russia, as well as between wild and cultivated types. The way in which we did so also provided an opportunity to scrutinize the different methods we employed.

### 4.1. Kale Variety Interrelatedness

Four different groups of kale were consistently identified across all analyses: (i) the curly kale varieties, (ii) Italian, (iii) American, and (iv) Russian varieties. Among the curly kales was a subgroup of varieties of East Frisian origin, but they did not build a monophylum. The Italian kales could be subdivided into Lacinato-type varieties and (Italian-derived) wild varieties. The wild, non-cabbage *Brassica* species were considered an outgroup. Our study gave insights into further interrelations of kale varieties.

We report high evidence for a common ancestry of kales “Lerchenzungen” and “Unterweser”. The SPLoSH value between the two was 797.1 cM (which is 66.2% of the genetic map), and they not only clustered together without exception, but they shared similar leaf morphology. Both have “Lerchenzungen”-type small, thin and finely-curled leaves, while “Unterweser” showed red coloring and a lower growth habit. In addition, the assumed Northern German origin of both varieties can be confirmed (“Lerchenzungen” is said to have been grown for a long time around the region of Hamburg, “Unterweser” between Bremen and Bremerhaven (cf. Supplementary Table S1)). They are found near to or within the East Frisian kales (Figure 5, Figure 6 and Figure S7B). “Unterweser” probably once emerged from crosses of “Lerchenzungen” and a red kale variety.

Further varieties for which our analyses gave evidence for a common ancestry were the German kales “Winnetou” and “Reflex”, which had the highest SPLoSH value here (1160.8 cM/96.3% of the map). Similarly, “Westländer Winter”, “Westländer Herbst”, and “Vitessa” seem to be highly related, given the remarkably high SPLoSH values for “Westländer Winter”/“Westländer Herbst” (957.4 cM), “Westländer Winter”/“Vitessa” (1041.5 cM), and “Westländer Herbst”/“Vitessa” (750.5 cM) (Supplementary Figure S1). Other groupings of varieties with SPLoSH values > 500 cM were (i) “Niedriger Grüner Krauser” and “Niedriger Grüner Feinstgekrauster”, (ii) “Halbhoher Grüner Mooskrauser” and “GDR kale”, as well as (iii) “Jellen” and “Lippischer Braunkohl”, each giving evidence for a common ancestry and corroborating results from the other methods. In addition, neighbor-net results indicated that the East Frisian kales “Schatteburg”, “Ostfriesische Palme”, and “Rote Palme” may have derived from similar origins.

Some kales treated as German, such as “Altmärker Braunkohl”, “Westerwoldse Grove”, and “Lippischer Braunkohl”, had a higher probability to be grouped within the East Frisian kales and might share ancestry with them (cf. Supplementary Figure S7), and, vice versa, the East Frisian kales “Neuefehn” and “Rosenweide” were more likely to belong to the German cluster. 

Our analyses highly suggest that the American kales have been bred from the headed and leafy cabbages and can now be considered as an own group (collards). There was only a little evidence for the presence of curly kale SNPs in the genomes of American kales (cf. Figure 4). Thus, they should be treated as var. *viridis* and not be grouped within the “sabellica” or “capitata” group [82]. Based on historical reports, American kale varieties are most likely derived from British kales and are grown in the coastal regions of the southeastern United States, especially North and South Carolina [82]. “Vates” and “Champion” share a common background (1087 cM SPLoSH value) with the latter being considered as an improved “Vates” variety [17]. The analyses gave evidence for also “Morris Heading” and “Georgia Southern” sharing a common background (810 cM). The curly kale “Olympic Red” consistently clustered within the curly varieties. Although being of American origin (bred in the USA by Nash Huber in Washington), its genetic background was clearly not inherited from collard types (as the other American kales) but instead from curly parent varieties. In neighbor-net and Bayesian analysis, it was neighbored to varieties “Winterbor” (869 cM) and “Starbor” (548 cM), suggesting genetic similarity and probably a common origin. In contrast, variety “Winter Red”—although said to be developed from Russian types (cf. Supplementary Table S1)—may rather be placed with the American collards.

The “Dutch kale” was conspicuous throughout all analyses. Although being a curly type kale it always clustered with the non-kale cabbages and near to (or within) the Italian kales, specifically “Negro Romano”, as seen from neighbor-net and Bayes tree (Figure 5 and Figure 6). The STRUCTURE plot and compoplot (Figure 4 and Figure S7) corroborated this by showing high probabilities of membership to non-kale *B. oleracea* and Italian clusters. Thus, it has to be doubted that the “Dutch kale” is solely of curly origin but instead shares some other alleles, possibly derived from an Italian gene pool.

“Siberian” and “Ragged Jack”, considered as Russian kales, clustered together with the wild *Brassicas* (or were found near to them), which is not surprising given their affiliation to *B. napus* (and not *B. oleracea*) and therefore are not to be classified as “true” kale varieties. The result of the Bayes tree (Figure 6), in which they were found among the curly kales, has to be considered an artefact of the long branch separating it from the other samples, due to the lack of B-genome markers in the SNP array and potentially inflated relatedness results from SPLoSH due to missing data (2.9 and 1.6% in filtered dataset, 1.7 and 0.7% in map dataset, respectively). 

For marrow-stem kale, both STRUCTURE and compoplot (Figure 4 and Figure S7) highly suggested a close genetic similarity with curly kales. This is remarkable since it is not reflected by its morphology. Additionally, in the Bayes tree it was, together with thousand-head kale, sister to the curly kale clade. Given these results, a common origin of these types should be further investigated. 

Regarding kohlrabi, our analyses reveal an Italian affiliation. This can be seen specifically in the compoplot of the filtered dataset (Supplementary Figure S7A). Only when using SPLoSH information was it constantly classified together with the other cabbages. Since the history and real origin of kohlrabi is largely unknown, this might be a hint of potential Mediterranean ancestry.

### 4.2. SPLoSH Information Improved Analyses of Kale Variety Interrelations

As a new approach for updating kale clustering information, we made use of shared haplotype information (SPLoSH) from SNP array data and applied this information to downstream analyses. To the best of our knowledge, information on shared haplotypes (or analogs of it) has not been previously used for the types of relationship evaluation presented here (PCA, neighbor networks, etc.). Referring to the different datasets used, the best results were generally achieved by using SPLoSH information. Similar results were observed with the filtered dataset being sufficiently useful in inferring structure among the samples, but the structure here was somewhat less clearly defined. Using a reduced number of SNPs in the map dataset was not sufficient to reliably infer kale interrelations, as there was generally a loss in cluster resolution. Additionally, the clear separation of curly kales in STRUCTURE plots dissolved, and the other clusters as well were far more heterogeneous (cf. Supplementary Figure S6). Furthermore, the Bayesian tree exhibited extraordinarily short branches (Supplementary Figure S9). For neighbor-net, no comprehensible distribution of samples among the network was visible, not to mention any sensible sample groups (cf. Supplementary Figure S8). Thus, the SNP selection for the map dataset seems to be of insufficient quality for inferring reliable kale interrelations. This loss of information in the smaller SNP set was compensated by including information about how those SNPs were linked via the use of SPLoSH information, resulting in sharper clustering. This method also increased the variance that was explained by the PCs in DAPC analysis by factor two (less than 40% with filtered dataset vs. more than 80% with SPLoSH information; cf. Figure 3). This improvement in clustering was made possible even with a relatively smaller number of SNPs (~1/3) compared to the filtered dataset, and while using a genetic map that was not designed for use with kale. Although missing data can influence relatedness results from SPLoSH, this did not seem to be relevant for our analysis, since by excluding samples with the highest missing data values, comparable results had been achieved for PCA and DAPC, for example (Supplementary Figure S10).

### 4.3. Curliness as a Criterion for Kale Variety Grouping

Nevertheless, the methods performed in this study differ in their usefulness for interrelation analyses within a species, such as *B. oleracea*. The variation that could be displayed by PCA and DAPC was limited, although DAPC was well suitable for separating the sample groups. Information about hybridization or introgression cannot be displayed by this method though. Nevertheless, similar to the report of Mabry, et al. [3], the analysis separated wild samples from cultivated samples. Since with Bayesian inference as a character-based method, it is impossible to process SPLoSH information in MrBayes, and also more informative in terms of reticulate evolution, uncertainties and conflicting signals in the data [83,84], networks are a better choice [85]. With the distance-based neighbor-net method [77], based on the neighbor joining algorithm [86], and a population structure analysis, a clear separation between “curly” and “non-curly” as well as “wild” and “cultivated” was obtained. Referring to Figure 4, purple and green coloring represent wild-type alleles, whereas red and blue coloring indicate derived alleles. Certainly, our analysis could not differentiate here further between American kale, wild Italian kale, and the non-kale *B. oleracea*. The STRUCTURE algorithm seemed to be at maximum resolution at *K* = 4 and no further structure was observed for higher values of *K* (data not shown). Hence, it was not possible to accurately delimit small sample groups with this analysis. Even though the compoplots from DAPC analysis (Supplementary Figure S7) suggested more detailed membership probabilities for several samples, it still seemed to be not very robust. Given different datasets, several varieties were assigned different membership probabilities. Although it was possible to apply the SPLoSH information here (which was not possible with STRUCTURE due to the nature of the SPLoSH data), the results, however, did not add new information (Supplementary Figure S7C), except for *B. carinata*, its *B. oleracea* background was seen here [40]. 

### 4.4. Limitations of Our Analyses

There were several limitations in this study that had an influence on the results and interpretations. First, the array itself may have posed a limitation. Evidence of this is in the low number of SNPs that were retained for analysis. Part of this is a reflection of the array being designed for *B. napus*, so already roughly half the SNPs from the *B. rapa* genome (together with *B. oleracea* the progenitor species of *B. napus* [40]) would be irrelevant to this study (since the SNPs from the *B. rapa* genome are less likely to work with kale). From the remaining SNPs from the *B. oleracea* genome, only a small percentage passed the strict quality control filters put in place. These filters were deemed necessary to ensure high quality and accurate results, but resulted in the retention of only a small percentage of the total SNPs available.

Second, to date, no integrated kale maps are available in the literature. Most of the *B. oleracea* or *B. napus* maps available are biparental. Integrated maps, however, would consider more SNPs and more recombination data. While applying the SPLoSH information for kale samples worked and led to higher quality results compared to just using raw SNPs, it suffered from the lack of a high-quality linkage map, since the map used here was not a kale map and included only 2668 SNPs. Because of the map limitations, the cM threshold values used were higher than what would be ideal for this sort of analysis and the lengths of shared haplotype length segments could be more precise. Thus, there is a need for a kale-specific linkage map with more SNPs for further comprehensive analyses of kale variety interrelations. 

Additionally, we did not have any comparison in the literature as to how well this approach worked compared to other approaches. In crops, SNP haplotype data usage has been limited. Examples have been presented by Toomajian, et al. [87], Cavanagh, et al. [88], Jordan, et al. [89], Poets, et al. [90], Hao, et al. [91], Coffman, et al. [92], Brinton, et al. [93], Roman and Houston [94], Wang, et al. [95], and Howard, et al. [58]. However, with this study, we could demonstrate that using haplotype data at least improves the results over the use of unlinked SNPs, even with the use of a suboptimal genetic map. If a more suitable map were available, one could expand the use of SPLoSH by looking for selection signatures across kale, within subpopulations, etc. Regarding the *Brassica* 60K array, so far only cluster files for the analysis of *B. oleracea* (C genome), *B. rapa* (A genome) and *B. napus* (A/C genome) are available [39]. To include other wild *Brassica* accessions (such as *B. montana*, *B. incana*, or *B. cretica*), a B genome cluster file would be needed [96]. Apart from that, the *Brassica* array is a valuable tool for studying genetic relationships in *B. oleracea* cultivars [39], as demonstrated for kale here. 

## 5. Conclusions

Four groups of kale were differentiated in this study: (i) the curly kale varieties, (ii) Italian, (iii) American, and (iv) Russian varieties. Our definition of kale includes the curly varieties as the “real” kales (“curly kale”), regardless of their origin (whether from East Frisia, other parts of Germany, or other countries). The group of Italian kales unites varieties of Lacinato type (palm kale, *B. oleracea* var. *palmifolia*) as well as others with an Italian background. The non-curled American kales should be correctly treated as Collards (var. *viridis*) and not be considered a kale group. The Russian kales are most distant from curly kale since they belong to *B. napus* var. *pabularia*, and although they share a (medium) curled leaf morphology with them, they should be considered a parallel evolutionary event to curly leaves. The use of SPLoSH information has been demonstrated as a valuable approach for analyzing kale variety interrelations and would likely have given even better results if an integrated kale map with more than 2668 SNPs could have been used. Constructing such a map is a crucial aspect for future research and would improve kale diversity analyses and enable other research as well. Currently, our results could contribute to a better understanding of the huge diversity of kale and its interrelations, and we encourage greater emphasis on this mostly overlooked species in crop research.

## Data Availability

The data presented in this study are available on request from the corresponding author.

## Supplementary Materials

The following supporting information can be downloaded at: https://www.mdpi.com/article/10.3390/genes13020232/s1, File S1: SNP datafile of filtered dataset, File S2: SNP datafile of map dataset, File S3: SPLoSH datafile, File S4: R-script for the analyses carried out, Figure S1: HeatMap visualizing the SPLoSH values, Figure S2: PCA results for the map dataset, Figure S3: PCA plots with all sample names, Figure S4: DAPC plots with all sample names, Figure S5: DAPC results for the map dataset, Figure S6: STRUCTURE results for the map dataset, Figure S7: DAPC compoplots, Figure S8: Neighbor-net for the map dataset, Figure S9: Bayes tree for the map dataset, Figure S10: DAPC plots excluding samples with high values of missing data, Table S1: Additional information on kale and cabbage varieties included in this study.
