# Supplementary material for: Different Shades of Kale—Approaches to Analyze Kale Variety Interrelations
Source: Genes (Basel). 2022 Jan 26;13(2):232. doi: 10.3390/genes13020232 (PMC8872201; doi:10.3390/genes13020232)
Supplement: Supplementary file 1 [file genes-13-00232-s001.zip › Supplementary Figure S2.pdf]

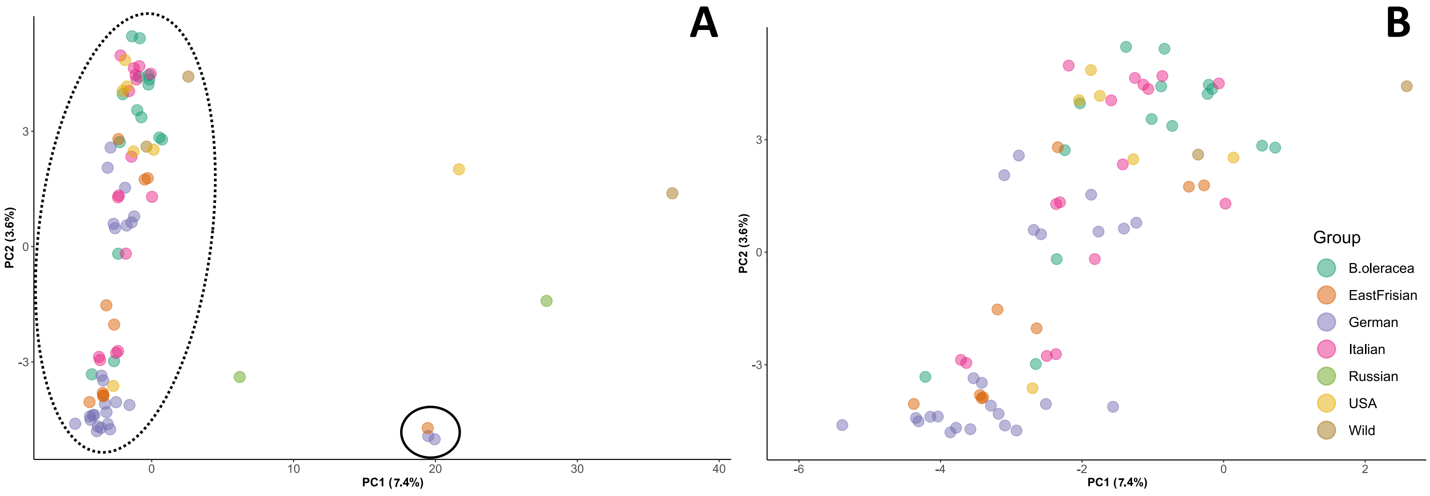

Supplementary Figure S2. Principal components score plot showing PC 1 and PC 2 of PCA results obtained with the SNPs from the map dataset. The proportion of the total variance that is explained by the first (PC 1) and second (PC 2) principal component is indicated in brackets. Each point represents one sample. No clear cluster pattern could be recognized. The same three curly kale varieties described in the text for the filtered dataset were separated (small circle), but, here, they appeared far more distant from the others on PC 1 than it was observed for the filtered dataset. A magnification of the remaining samples (dotted circle) could not reveal any further clustering according to PC 1 and PC 2 (Figure part B).
