# Supplementary material for: Different Shades of Kale—Approaches to Analyze Kale Variety Interrelations
Source: Genes (Basel). 2022 Jan 26;13(2):232. doi: 10.3390/genes13020232 (PMC8872201; doi:10.3390/genes13020232)
Supplement: Supplementary file 1 [file genes-13-00232-s001.zip › Supplementary Figure S4.pdf]

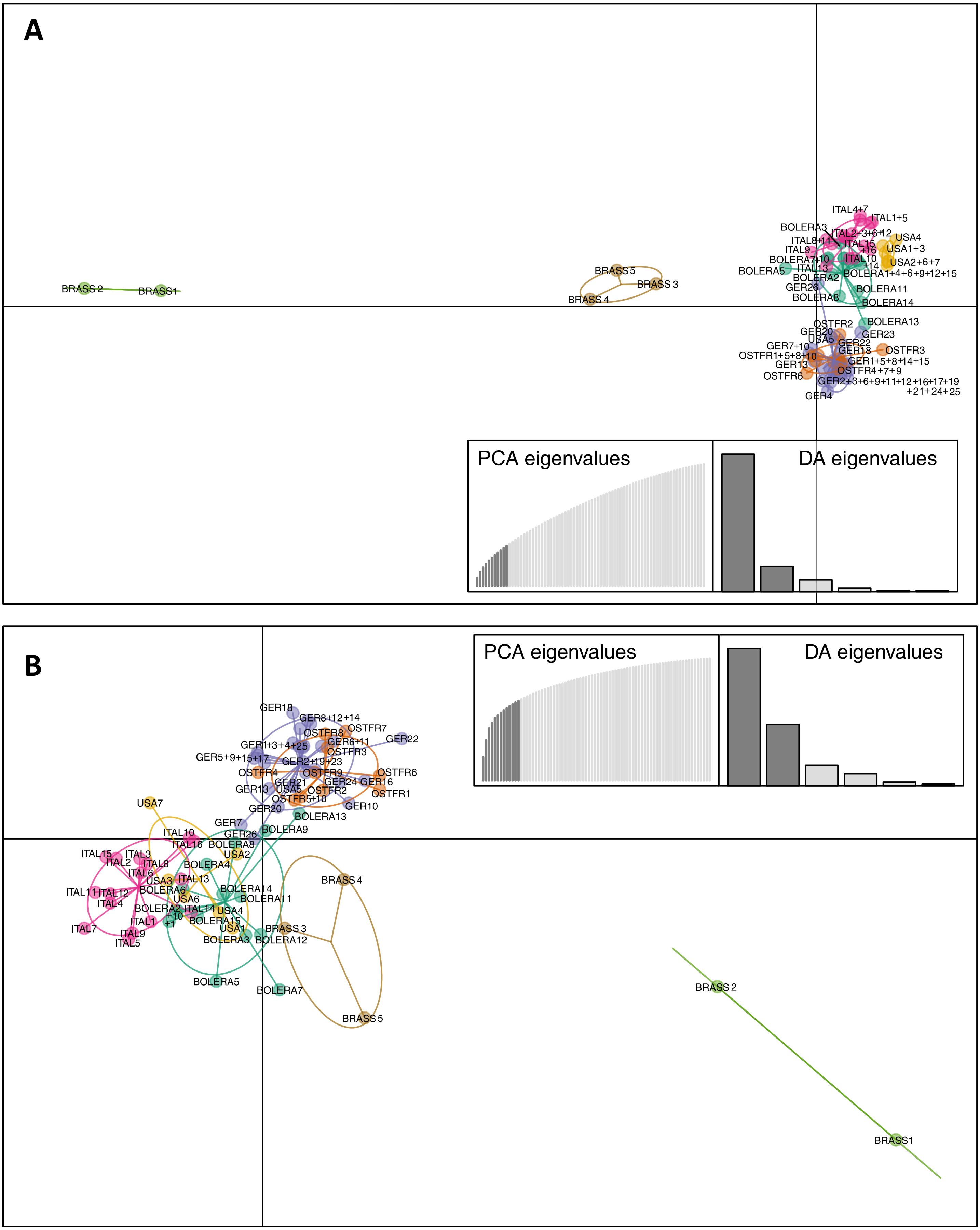

Supplementary Figure S4.(continued on next page)

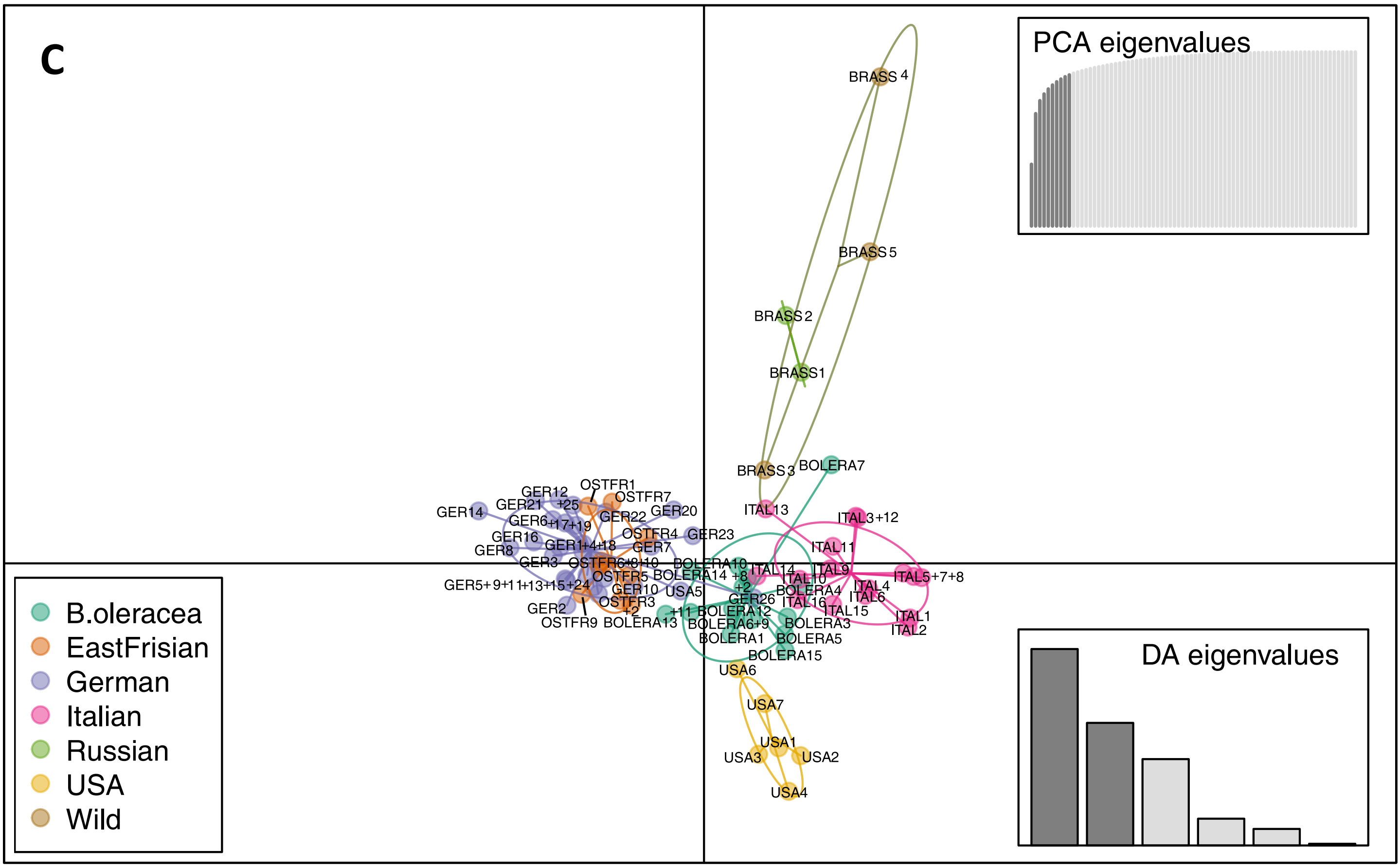

Supplementary Figure S4. Discriminant analysis of principal components (DAPC). Scatterplot (with sample names) showing the first two principal components for the samples obtained from (**A**) SNP data of the filtered dataset, (**B**) the SNPs of the map dataset, and (**C**) using the SPLoSH information. Dots represent individual samples, clusters are marked with ellipses. Graphs of the PCA and DA eigenvalues retained are shown. For details see text.
