# Supplementary material for: Different Shades of Kale—Approaches to Analyze Kale Variety Interrelations
Source: Genes (Basel). 2022 Jan 26;13(2):232. doi: 10.3390/genes13020232 (PMC8872201; doi:10.3390/genes13020232)
Supplement: Supplementary file 1 [file genes-13-00232-s001.zip › Supplementary Figure S5.pdf]

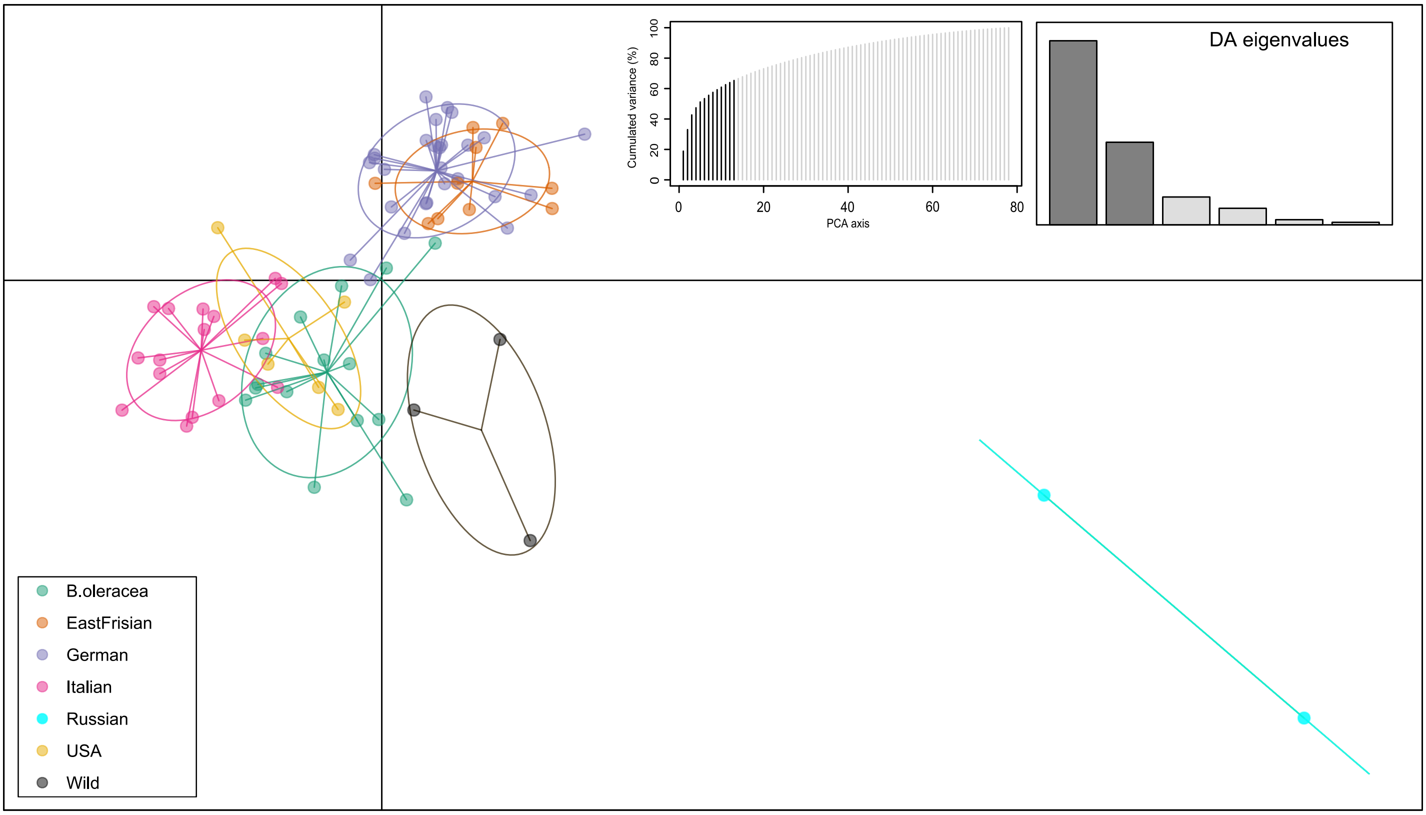

Supplementary Figure S5. Discriminant analysis of principal components (DAPC). Scatterplot showing the first two principal components for the samples obtained from the map dataset SNPs. Dots represent individual samples, clusters are marked with ellipses. Graphs of the PCA and DA eigenvalues retained are shown. Compared to the filtered dataset, the patterns became blurrier, since there was no clear separation between American and Italian kales and non-kale cabbages (*B. oleracea*).
