# Supplementary material for: Different Shades of Kale—Approaches to Analyze Kale Variety Interrelations
Source: Genes (Basel). 2022 Jan 26;13(2):232. doi: 10.3390/genes13020232 (PMC8872201; doi:10.3390/genes13020232)
Supplement: Supplementary file 1 [file genes-13-00232-s001.zip › Supplementary Figure S6.pdf]

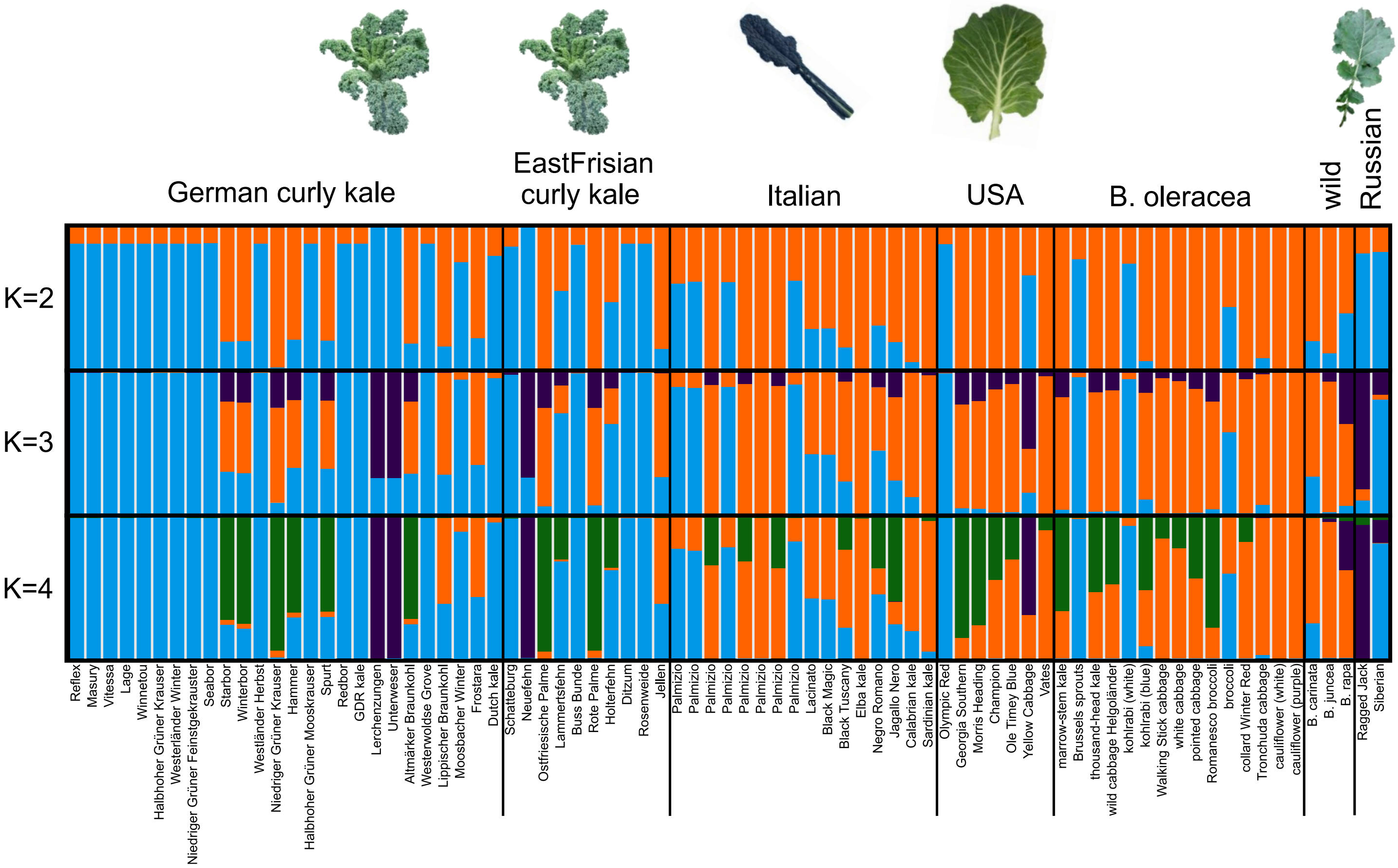

Supplementary Figure S6. STRUCTURE plot for 79 kale and cabbage varieties from different origins for $K = 2$-4, based on the SNP data from the map dataset. Each column represents one variety, colors indicate the proportion of membership to different clusters. Each figure is a combination of 20 replicates. Here, the groups were less clearly separated and more heterogeneous, compared to the filtered dataset.
