# Supplementary material for: Different Shades of Kale—Approaches to Analyze Kale Variety Interrelations
Source: Genes (Basel). 2022 Jan 26;13(2):232. doi: 10.3390/genes13020232 (PMC8872201; doi:10.3390/genes13020232)
Supplement: Supplementary file 1 [file genes-13-00232-s001.zip › Supplementary Figure S7.pdf]

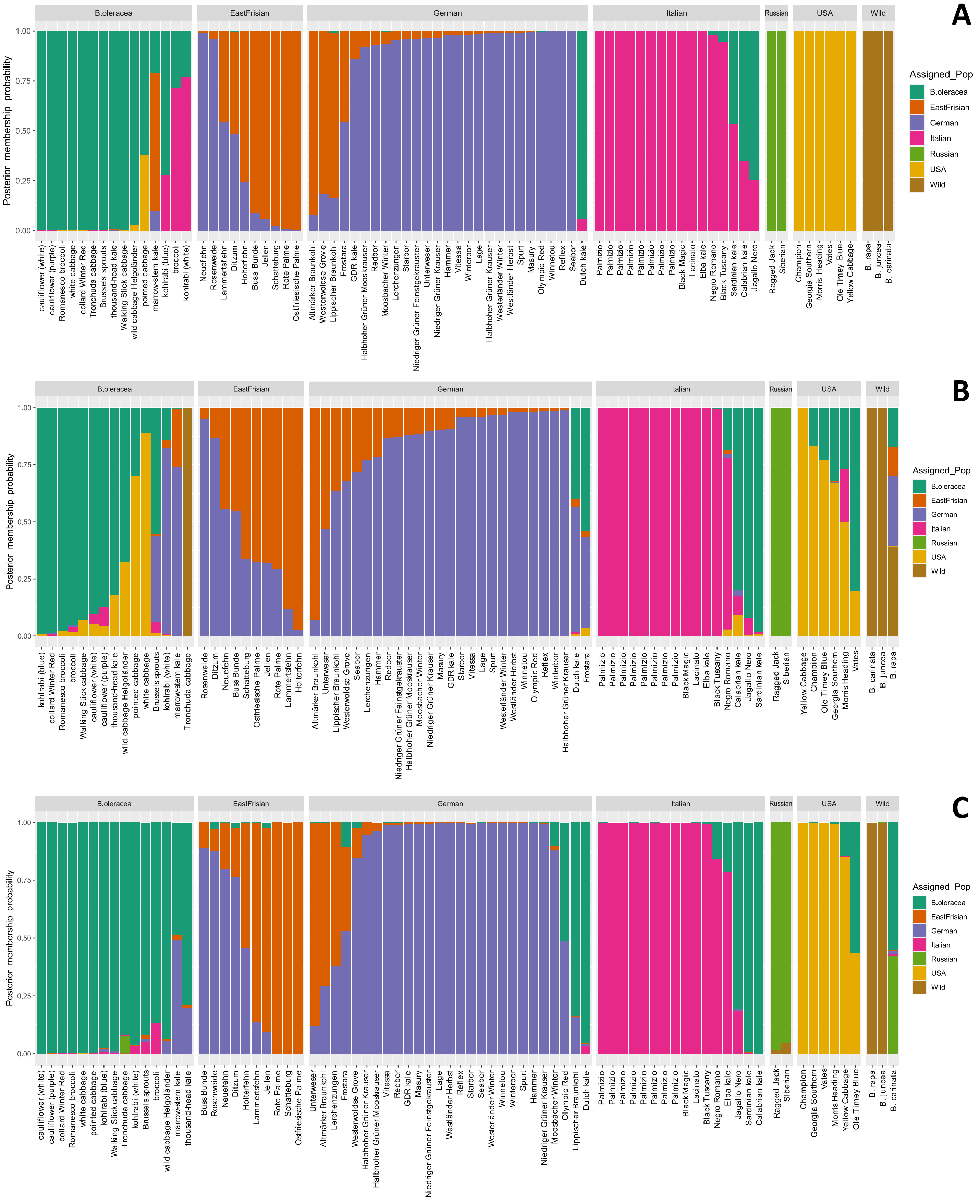

Supplementary Figure S7. DAPC compoplot with membership probabilities for the kale and cabbage samples for (**A**) the filtered dataset, (**B**) the map dataset, and (**C**) the SPLoSH information. Each column refers to one variety, colors are proximities of varieties to different clusters. Varieties are ordered within each group according to proportion of coloring.
