# Supplementary material for: Different Shades of Kale—Approaches to Analyze Kale Variety Interrelations
Source: Genes (Basel). 2022 Jan 26;13(2):232. doi: 10.3390/genes13020232 (PMC8872201; doi:10.3390/genes13020232)
Supplement: Supplementary file 1 [file genes-13-00232-s001.zip › Supplementary Figure S8.pdf]

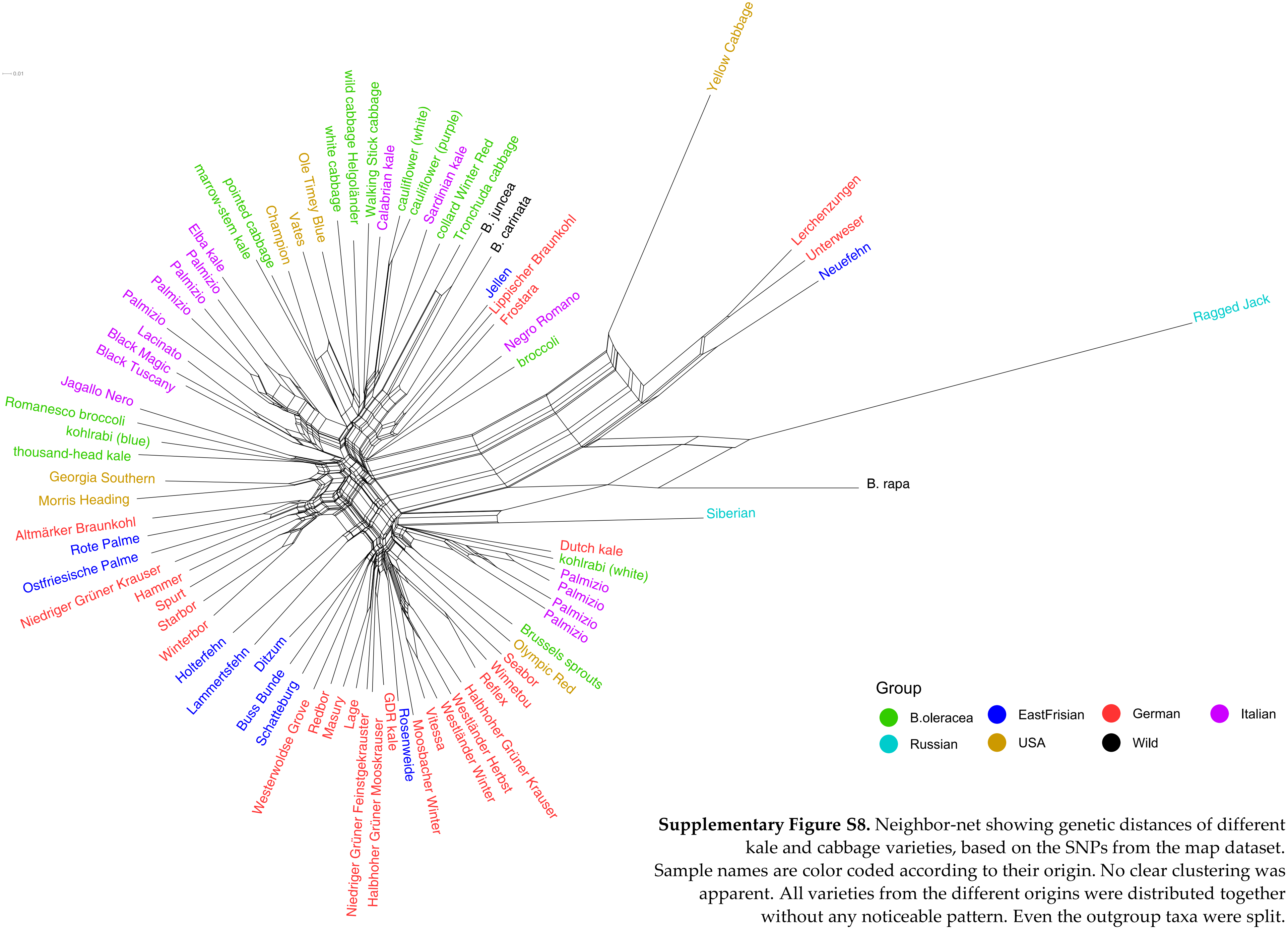

Supplementary Figure S8. Neighbor-net showing genetic distances of different kale and cabbage varieties, based on the SNPs from the map dataset. Sample names are color coded according to their origin. No clear clustering was apparent. All varieties from the different origins were distributed together without any noticeable pattern. Even the outgroup taxa were split.
