# Supplementary material for: Different Shades of Kale—Approaches to Analyze Kale Variety Interrelations
Source: Genes (Basel). 2022 Jan 26;13(2):232. doi: 10.3390/genes13020232 (PMC8872201; doi:10.3390/genes13020232)
Supplement: Supplementary file 1 [file genes-13-00232-s001.zip › Supplementary Table S1.pdf]

Supplementary Table S1. Additional information on kale and cabbage varieties included in this study.

Abbreviation	Variety name		Group (origin)	Phenotypic grouping	Seed supplier	Additional information on varieties
GER 1	Halbhoher Grüner Krauser	*[1]	German	curly	Bruno Nebelung GmbH, Germany	
GER 2	Lerchenzungen	*	German	curly	Bruno Nebelung GmbH, Germany	150 yr old variety
GER 3	Vitessa	#	German	curly	N.L.Chrestensen Erfurt, Germany	
GER 4	Lage Fijngekrulde	*	German	curly	N.L.Chrestensen Erfurt, Germany	
GER 5	Niedriger Grüner Krauser	*	German	curly	PNOS, Poland	
GER 6	Niedriger Grüner Feinstgekrauster		German	curly	IPK Gatersleben, Germany	
GER 7	Frostara	*	German	curly	Bruno Nebelung GmbH, Germany	
GER 8	Winnetou F1	*	German	curly	Bruno Nebelung GmbH, Germany	
GER 9	Winterbor F1	*	German	curly	Samen Gernand GmbH, Germany	
GER 10	Westerwoldse Grove		German	curly	IPK Gatersleben, Germany	
GER 11	Westländer Winter	*	German	curly	Van Hemert & Co, the Netherlands	
GER 12	Westländer Herbst	*	German	curly	Buzzy Seeds, the Netherlands	
GER 13	Redbor F1	*	German	curly	Bruno Nebelung GmbH, Germany	'Winterbor'-developed
GER 14	Reflex F1	*	German	curly	Dürr Samen S. Schwenk, Germany	'Westländer' type
GER 15	Starbor F1	*	German	curly	Thompson & Morgan, UK	
GER 16	Seabor F1	#	German	curly	Hazzard's Seeds, USA	
GER 17	Spurt		German	curly	IPK Gatersleben, Germany	
GER 18	Hammer	#	German	curly	IPK Gatersleben, Germany	
GER 19	Masury		German	curly	IPK Gatersleben, Germany	
GER 20	Moosbacher Winter		German	curly	IPK Gatersleben, Germany	originated in DDR/East Germany
GER 21	Halbhoher Grüner Mooskrauser		German	curly	IPK Gatersleben, Germany	
GER 22	Altmärker Braunkohl	*	German	curly	Bruno Nebelung GmbH, Germany	native to Altmark region
GER 23	Lippischer Braunkohl	*	German	curly	IPK Gatersleben, Germany	native to Lippe region
GER 24	Unterweser		German	curly	IPK Gatersleben, Germany	collected near Brake/Unterweser
GER 25	GDR kale		German	curly	IPK Gatersleben, Germany	originated in German Democratic Republic (GDR)/East Germany
GER 26	Dutch kale		German	curly	IPK Gatersleben, Germany	collected in the Netherlands
OSTFR 1	Buss Bunde		EastFrisian	curly	R. Lühring, farmer, Germany	old Northern German landrace
OSTFR 2	Ditzum		EastFrisian	curly	R. Lühring, farmer, Germany	old Northern German landrace
OSTFR 3	Jellen		EastFrisian	curly	R. Lühring, farmer, Germany	old Northern German landrace
OSTFR 4	Rosenweide		EastFrisian	curly	R. Lühring, farmer, Germany	old Northern German landrace
OSTFR 5	Rote Palme		EastFrisian	curly	R. Lühring, farmer, Germany	old Northern German landrace
OSTFR 6	Schatteburg		EastFrisian	curly	R. Lühring, farmer, Germany	old Northern German landrace
OSTFR 7	Holterfehn		EastFrisian	curly	R. Lühring, farmer, Germany	old Northern German landrace
OSTFR 8	Lammertsfehn		EastFrisian	curly	R. Lühring, farmer, Germany	old Northern German landrace
OSTFR 9	Neuefehn		EastFrisian	curly	R. Lühring, farmer, Germany	old Northern German landrace
OSTFR 10	Ostfriesische Palme	*	EastFrisian	curly	R. Lühring, farmer, Germany	developed from 'Lerchenzungen' old Northern German landrace
ITAL 1-8	Palmizio Senza Testa	#	Italian	Lacinato-type	Thompson & Morgan, UK	
ITAL 9	Black Tuscany (Nero di Toscana)	*	Italian	Lacinato-type	Thompson & Morgan, UK	
ITAL 10	Negro Romano		Italian	Lacinato-type	R. Lühring, farmer, Germany	
ITAL 11	Black Magic	*	Italian	Lacinato-type	Gärtner Pötschke GmbH, Germany	'Black Tuscany'-developed[2]
ITAL 12	Lacinato		Italian	Lacinato-type	Gartencenter Schenker, Germany	
ITAL 13	Jagallo Nero		Italian	Lacinato-type	Chiltern Seeds Limited, UK	'Negro Romano'-developed
ITAL 14	Sardinian kale		Italian	wild, non-Lacinato	IPK Gatersleben, Germany	native to Bari (Sardinia island)
ITAL 15	Elba kale		Italian	wild, non-Lacinato	IPK Gatersleben, Germany	native to Elba island
ITAL 16	Calabrian kale		Italian	wild, non-Lacinato	R. Lühring, farmer, Germany	native to Calabria
USA 1	Champion		American	non-curled collards	Sustainable Seed Company, USA	improved 'Vates' variety
USA 2	Georgia Southern		American	non-curled collards	Sustainable Seed Company, USA	cultivated since 1880
USA 3	Morris Heading		American	non-curled collards	Sustainable Seed Company, USA	Heirloom variety
USA 4	Vates		American	non-curled collards	Sustainable Seed Company, USA	Heirloom variety
USA 5	Olympic Red		American	curly	Restoration Seeds, USA	farmer-bred curly kale[3]
USA 6	Ole Timey Blue		American	non-curled collards	Restoration Seeds, USA	Heirloom from Cherokee people
USA 7	Yellow Cabbage		American	non-curled collards	Restoration Seeds, USA	Heirloom, Carolina Cabbage Collard
BRASS 1	Ragged Jack (Red Russian)	‡	Russian	lobed / frilled	IPK Gatersleben, Germany	pre-1885 Heirloom
BRASS 2	Siberian		Russian	lobed / frilled	Exotic-Samen W. Meier, Germany	
BRASS 3	*Brassica carinata*		wild Brassica		Dreschflegel GbR, Germany	'Carina'
BRASS 4	*Brassica rapa*		wild Brassica		Bruno Nebelung GmbH, Germany	subsp. *pekinensis* 'Michihili' (Chinese cabbage)
BRASS 5	*Brassica juncea*		wild Brassica		Dreschflegel GbR, Germany	var. *rugosa* 'Red Giant'

Supplementary Table S1. (continued)

Abbreviation	Variety name		Group (origin)	Phenotypic grouping	Seed supplier	Additional information on varieties
BOLERA 1	cauliflower white ‘Optimist F1’ (*B. oleracea* var. *botrytis*)[4]	*	non-kale *B. oleracea*		Carl Sperling & Co. GmbH, Germany	
BOLERA 2	cauliflower purple (*B. oleracea* var. *botrytis*)		non-kale *B. oleracea*		Dürr Samen S. Schwenk, Germany	
BOLERA 3	romanesco broccoli (*B. oleracea* convar. *botrytis* var. *botrytis*)		non-kale *B. oleracea*		Magic Garden Seeds, Germany	Italian derived cauliflower speciality
BOLERA 4	broccoli ‘Calabrese natalino’ (*B. oleracea* var. *italica*)		non-kale *B. oleracea*		Gärtner Pötschke GmbH, Germany	
BOLERA 5	white cabbage ‘Brunswijker’ (*B. oleracea* var. *alba*)	*	non-kale *B. oleracea*		Bruno Nebelung GmbH, Germany	
BOLERA 6	pointed cabbage ‘Express’ (*B. oleracea* var. *alba* subvar. *conica*)	*	non-kale *B. oleracea*		Quedlinburger Saatgut, Germany	
BOLERA 7	Tronchuda cabbage (*B. oleracea* var. *costata*)		non-kale *B. oleracea*		Magic Garden Seeds, Germany	
BOLERA 8	Brussels sprouts ‘Hilds Ideal’ (*B. oleracea* var. *gemmifera*)	#	non-kale *B. oleracea*		Centor-Warenhandels GmbH, Germany	
BOLERA 9	kohlrabi white ‘Delikateß weißer’ (*B. oleracea* var. *gongylodes*)	*	non-kale *B. oleracea*		Bruno Nebelung GmbH, Germany	
BOLERA 10	kohlrabi blue ‘Delikateß blauer’ (*B. oleracea* var. *gongylodes*)	*	non-kale *B. oleracea*		N.L.Chrestensen Erfurt, Germany	
BOLERA 11	wild cabbage Helgoländer (*B. oleracea* L.)		non-kale *B. oleracea*		Botanical Garden Oldenburg, Germany	collected in the cliffs of Helgoland island
BOLERA 12	Walking Stick cabbage ‘Jersey kale’ (*B. oleracea* var. *medullosa*)		non-kale *B. oleracea*		Thompson & Morgan, UK	native to the Channel Island Jersey
BOLERA 13	marrow-stem kale ‘Westfälischer Furchenkohl’ (*B. oleracea* var. *medullosa*)		non-kale *B. oleracea*		Bruno Nebelung GmbH, Germany	
BOLERA 14	thousand-head kale ‘Dwarf Canson’ (*B. oleracea* var. *ramosa*)		non-kale *B. oleracea*		IPK Gatersleben, Germany	originally from England
BOLERA 15	collard Winter Red (unknown)		non-kale *B. oleracea*		IPK Gatersleben, Germany	

[1] Varieties marked with an asterix (*) are registered in the EU Plant Variety Database. Those with an (#) were formerly registered and now deleted. Varieties marked with an (‡) are listed in the UK National List of varieties of Vegetable Plant Species.
[2] Developed by botanist James Wong, London, UK.
[3] Developed by farmer Nash Huber, Washington, USA.
[4] We generally followed the classification by Gladis and Hammer [65] but shortened the intraspecific grouping for better reading.
