# Supplementary figures and images for: Different Shades of Kale—Approaches to Analyze Kale Variety Interrelations
Source: Genes (Basel). 2022 Jan 26;13(2):232. doi: 10.3390/genes13020232 (PMC8872201; doi:10.3390/genes13020232)

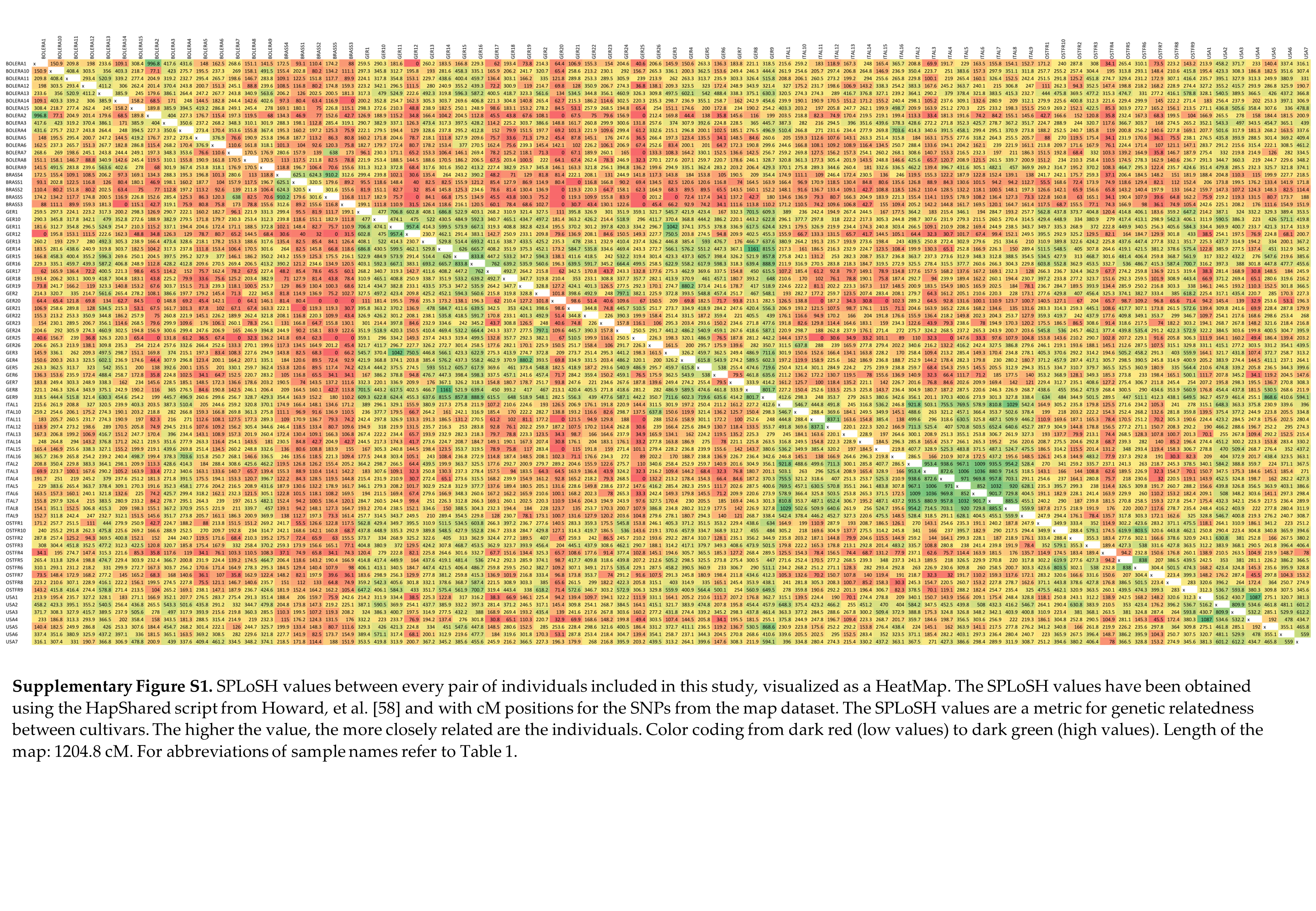

Supplement: Supplementary file 1 [file genes-13-00232-s001.zip › Supplementary Figure S1.png]

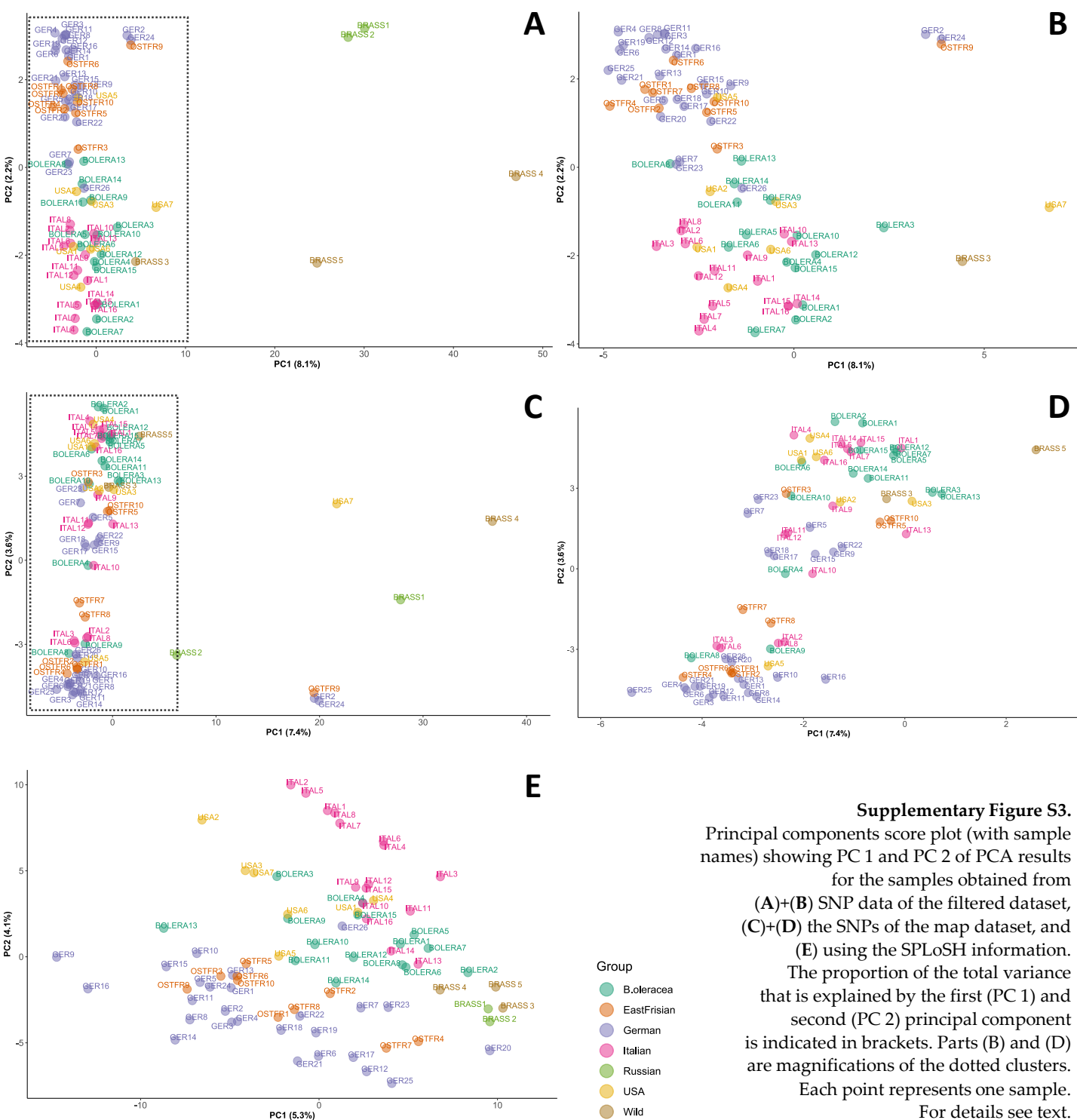

Supplement: Supplementary file 1 [file genes-13-00232-s001.zip › Supplementary Figure S3.pdf]

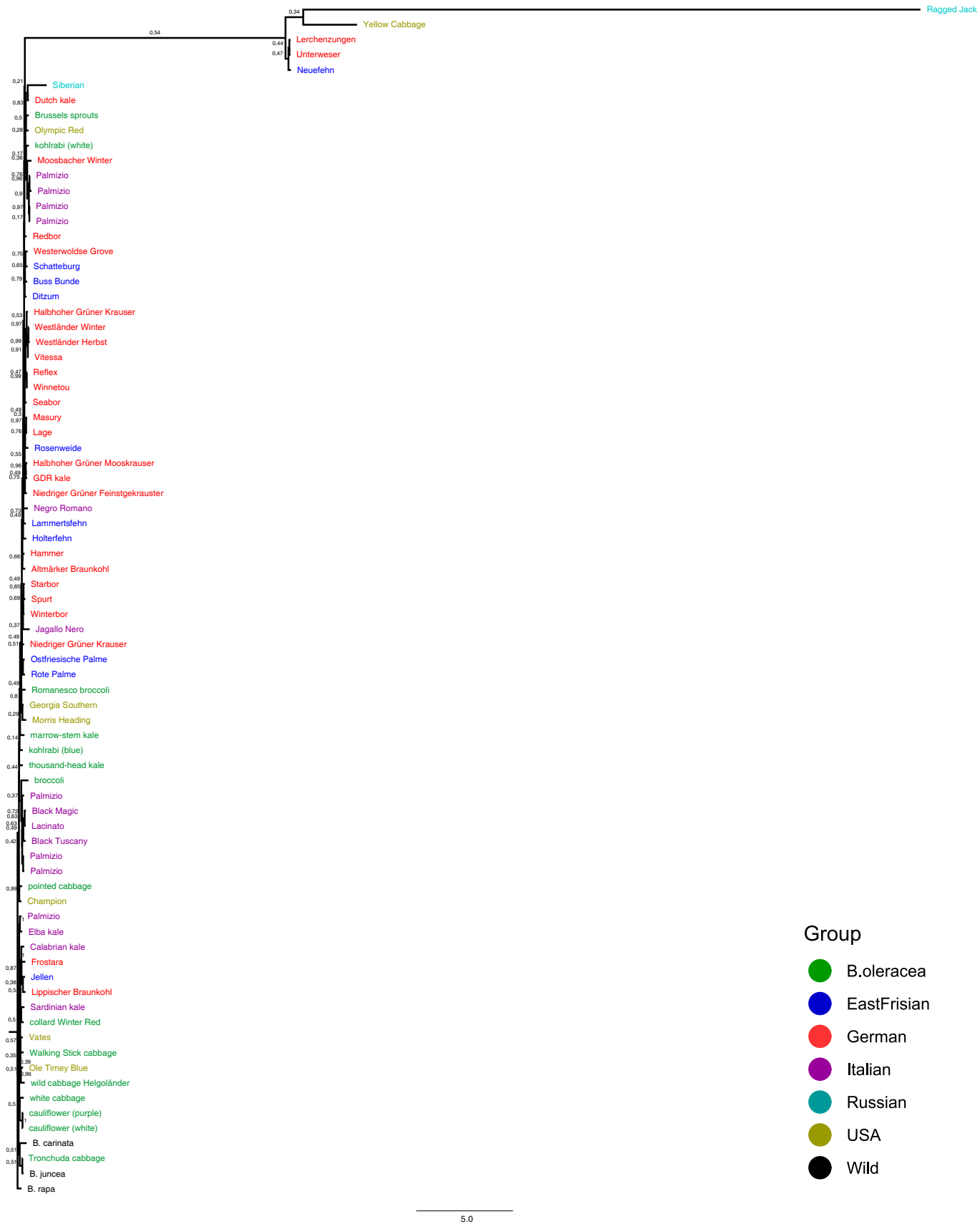

Supplement: Supplementary file 1 [file genes-13-00232-s001.zip › Supplementary Figure S9.pdf]
